# The Role of microRNAs in Epithelial Ovarian Cancer Metastasis

**DOI:** 10.3390/ijms21197093

**Published:** 2020-09-25

**Authors:** Vu Hong Loan Nguyen, Chenyang Yue, Kevin Y. Du, Mohamed Salem, Jacob O’Brien, Chun Peng

**Affiliations:** 1 Department of Biology, York University, Toronto, ON M3J 1P3, Canada; hlnguyen@my.yorku.ca (V.H.L.N.); chenyangyue1993@gmail.com (C.Y.); kevin.yc.du@gmail.com (K.Y.D.); Mohamed.salem@cshs.org (M.S.); jaobr@my.yorku.ca (J.O.); 2 Centre for Research in Biomolecular Interactions, York University, Toronto, ON M3J 1P3, Canada

**Keywords:** epithelial ovarian cancer, metastasis, microRNAs (miRNAs)

## Abstract

Epithelial ovarian cancer (EOC) is the deadliest gynecological cancer, and the major cause of death is mainly attributed to metastasis. MicroRNAs (miRNAs) are a group of small non-coding RNAs that exert important regulatory functions in many biological processes through their effects on regulating gene expression. In most cases, miRNAs interact with the 3′ UTRs of target mRNAs to induce their degradation and suppress their translation. Aberrant expression of miRNAs has been detected in EOC tumors and/or the biological fluids of EOC patients. Such dysregulation occurs as the result of alterations in DNA copy numbers, epigenetic regulation, and miRNA biogenesis. Many studies have demonstrated that miRNAs can promote or suppress events related to EOC metastasis, such as cell migration, invasion, epithelial-to-mesenchymal transition, and interaction with the tumor microenvironment. In this review, we provide a brief overview of miRNA biogenesis and highlight some key events and regulations related to EOC metastasis. We summarize current knowledge on how miRNAs are dysregulated, focusing on those that have been reported to regulate metastasis. Furthermore, we discuss the role of miRNAs in promoting and inhibiting EOC metastasis. Finally, we point out some limitations of current findings and suggest future research directions in the field.

## 1. Introduction

Ovarian cancer is the fifth leading cause of cancer-related deaths in females [1]. Based on the cell origin where ovarian tumors arise, ovarian cancer is classified into three categories: epithelial, germ cell, and stromal ovarian cancer. Several types of extremely rare ovarian cancer, such as small cell carcinoma and sarcomas, have also been reported [2]. Among them, epithelial ovarian cancer (EOC) accounts for more than 85% of ovarian cancer cases and is responsible for most ovarian cancer-related deaths [3]. EOC is further grouped into five different histological subtypes, including high-grade serous carcinomas (HGSC), low-grade serous carcinomas (LGSC), endometrioid carcinomas (EC), clear cell carcinomas (CCC), and mucinous carcinomas (MC) [3]. Though the morbidity of ovarian cancer is lower than that in endometrial and cervical cancers, it has the highest mortality rate among gynecological cancers [1]. The five-year survival rate of EOC is less than 45% [4], and relapse and poor prognosis occur in 80% of patients with advanced stages [5,6]. EOC is difficult to detect at the early stages as there are no effective screening methods and the presenting symptoms are vague. Therefore, patients are often diagnosed at the advanced stages when the tumor metastasis is already taking place [5].

MicroRNAs (miRNAs) are small non-coding RNAs that regulate gene expression within cells [7,8]. Studies have shown that 30–60% of human protein-coding genes are regulated by miRNAs [9]. Through regulation of the target gene expression, miRNAs are reported to control many biological processes, including proliferation, differentiation, cell cycle progression, apoptosis, and immune response [10]. Aberrant expression of miRNAs is implicated in many diseases, including cancer. Studies have demonstrated that miRNAs are involved in the progression of EOC [11,12]. Their levels are up- or down-regulated in EOC tumors and/or patient plasma samples, and their abnormal expression is highly associated with EOC metastasis [11,13]. In this review, we provide a brief overview of the biogenesis and mechanisms of actions of miRNAs and metastasis in EOC. We then discuss the dysregulation of miRNAs in EOC and the roles of miRNAs in promoting or suppressing cellular processes related to metastasis. Finally, we point out some limitations of current studies and suggest future research directions.

## 2. Overview of miRNAs

Depending on the genomic location, miRNAs can generally be classified as intragenic or intergenic. Intragenic miRNAs, which account for approximately 50% of the annotated miRNAs in humans, are generated from a host gene, mainly within the introns of protein-coding genes [14]. Expression of intragenic miRNAs is usually coupled with their host genes, while the transcription of intergenic miRNAs is directed by their own promoters [15,16]. However, it has also been reported that some intragenic miRNAs are transcribed independently of their host genes, generating pri-miRNA that also undergoes splicing [17].

The transcription of the primary miRNA (pri-miRNA) by RNA polymerase II is the first step of canonical miRNA biogenesis. Depending on the source of the miRNA, the pri-miRNA may be the mRNA of the host gene or an independent transcription unit, both containing a 5′ m7G cap and a 3′ poly-A tail [18,19]. Subsequently, pri-miRNAs undergo extensive processing within the nucleus. In the canonical pathway, the pri-miRNAs are converted into ~70 nt hairpin miRNA precursors (pre-miRNAs) via the microprocessor complex, which consists of an RNase III endonuclease, DROSHA, and the double-stranded-RNA-binding protein, DGCR8 (DiGeorge syndrome Critical Region 8) [20,21,22]. DGCR8 acts as the regulatory subunit that recognizes specific motifs within pri-miRNAs while DROSHA functions as the catalytic subunit. The pre-miRNA is then exported to the cytoplasm via a member of the nuclear transport receptor family, Exportin 5 (XPO5), together with Ran-GTP [20,23,24]. In the non-canonical pathway, precursor miRNAs are processed independently of the DROSHA/DGCR8 complex in the nucleus and exported outside the nucleus via Exportin 1 [25,26].

In the cytoplasm, the pre-miRNAs are further processed into mature miRNAs by another RNase III endonuclease, Dicer [20,27]. Dicer cleaves pre-miRNA near the terminal loop, resulting in a short, on average 22 nt, RNA duplex [27,28,29]. Two mature miRNAs, originating from the 5′ and 3′ ends of the miRNA duplex and denoted with the postfix -5p and -3p, respectively, can be generated through an unwinding process, guided by Argonaute (AGO) [30,31]. The miRNA duplex is unwound in an ATP-dependent manner and directly interacts with AGO via the 5′ and 3′ nucleotides of the miRNA, creating a stable association [32,33,34]. The strand that is incorporated into the miRNA-induced silencing complex (miRISC) is referred to as the “guide strand” and the other strand, known as the “passenger strand”, is degraded [35,36]. Four AGO proteins (AGO1–4) have been characterized in humans and they are all capable of interacting with miRNAs [37].

**Figure 1:**
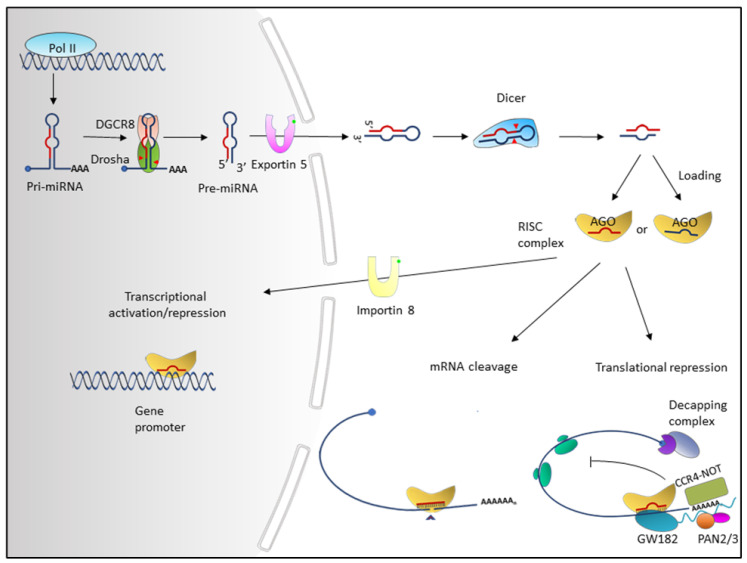
Biogenesis and functions of miRNA. MicroRNAs (miRNAs) are usually transcribed by polymerase II in the nucleus to generate primary microRNAs (pri-miRNA) transcripts. In the canonical pathway, the maturation of miRNAs is then performed by the microprocessor complex comprising of DROSHA, RNase III, and DCGR8. The microprocessor complex cleaves both strands of pri-miRNAs near the base of the primary stem loop, generating pre-miRNAs. Following cleavage, pre-miRNAs are actively transported from the nucleus to the cytoplasm by the Exportin 5/Ran-GTP complex. In the cytoplasm, RNase III endonuclease Dicer recognizes and cleaves pre-miRNA near the terminal loop, releasing a small RNA duplex. Subsequently, either strand (-5p or -3p) of the miRNA duplex is loaded into an AGO protein to form a miRNA-induced silencing complex (miRISC). Functionally, miRNAs direct the miRISC complex to target genes and modulate its expression by promoting either mRNA degradation and/or translation inhibition in the cytoplasm. The AGO protein of miRISC complex binds GW182 family proteins, which serve as scaffolds for multiple proteins including PAN2/3, and CCR4-NOT complexes. While the miRISC complex hinders the binding of eIF4F complex, PAN2/3 and CCR4-NOT mediate the poly(A) deadenylation of target mRNAs. The target mRNA is further decapped by a decapping complex and subjected to degradation via the exoribonuclease XRN1. In addition, the miRISC complex can be transported into the nucleus via Importin 8/RAN-GTP complex and binds to target gene promoters to regulate its transcription.

In most reported cases, miRNAs repress their target genes at the post-transcriptional level [38]. They bind to partial complementary sequences in the 3′ untranslated region (UTR) of their target mRNAs, called the miRNA response element (MRE), inducing mRNA degradation and inhibiting translation initiation [39]. The AGO-bound miRNA and MRE interactions act as a guide to enable additional miRISC components to regulate target mRNA stability and protein output. GW182, which interacts directly with AGO, is crucial in localizing the poly-A tail of target mRNA to miRISC, as well as in the recruitment of deadenylase and decapping complexes [40]. PAN2/3 and CCR4/NOT deadenylase complexes are recruited via interaction with tryptophan motifs of GW182 [41,42]. Subsequently, mRNA poly(A) deadenylation is initiated by PAN2/3 and completed by CCR4/NOT complexes [41,42], followed by m7G decapping, facilitated by decapping protein 2 (DCP2) and associated proteins [43]. The unstable deadenylated and decapped mRNA can then be degraded by 5′–3′ exoribonuclease 1 (XRN1) [44]. In addition, miRNAs repress the translation initiation process through the release of eukaryotic initiation factors, eIF4A1 and eIF4A2, from the target mRNAs; as such, in some cases, miRNA can affect protein levels while leaving mRNA stability unaffected [45] (Figure 1).

More recently, it has been shown that miRNAs have binding sites on other regions of the mRNAs, including coding regions and 5′ UTR, or on DNA promoter regions [46]. Interestingly, it was found that the binding of miRNA to the coding regions or 5′ UTR of mRNA exerts a silencing effect on gene expression while the interaction of miRNA with the promoter region could induce transcription [47,48,49]. Conversely, there is some evidence supporting miRNAs’ role in promoting translation under specific conditions [50,51].

## 3. Ovarian Cancer Metastasis

Metastasis is a complex multistep process in which cancer cells disseminate from primary tumors and start new tumors at different sites in the body. This process is regulated by a specific set of genes and signaling pathways. EOC cells mainly metastasize through the transcoelomic pathway [52], in which cells disseminate from the primary EOC tumor by undergoing epithelial-to-mesenchymal transition (EMT) [52] and float freely as spheroids in the ascitic fluid within the peritoneal cavity. The metastatic cells then attach onto the mesothelium lining or invade deeper into the peritoneal organs [53]. In addition, metastatic ovarian cancer cells can transit in the blood or lymph vessels and undergo extravasation to establish new tumors in hematogenous and lymphatic metastasis [53,54]. EOC metastasis to secondary sites accounts for approximately 90% of all ovarian cancer deaths [53]. Therefore, understanding the underlying mechanisms of EOC metastasis could lead to the development of more effective therapeutic tools.

EMT is a biological process which is activated during normal embryonic and organ development, as well as tissue repair [55]. The role of EMT in tumor metastasis has been established in many types of cancers, including EOC [56,57,58]. In EMT, epithelial cells undergo phenotypic alterations through the loss of cell polarity, cell–cell attachment, and gain mesenchymal phenotypes, such as fibroblastoid morphology with increased migratory and invasive properties. EMT is a critical step in ovarian cancer metastasis [59]. Downregulation of epithelial cadherin (E-cadherin, CDH1) and upregulation of mesenchymal neural cadherin (N-cadherin, CDH2) are key elements of EMT. E-cadherin is a transmembrane glycoprotein that associates with β-catenin at the adherens junctions [59]. Loss of E-cadherin results in the destabilization of adherens junctions, promoting cell migration, invasion, and metastasis. E-cadherin expression is repressed directly by many transcription factors, including Snail (SNAI1), Slug (SNAI2), and zinc finger E-box binding homeobox (ZEB)1 and ZEB2, and indirectly by TWIST and TCF4 [60,61]. In addition, Vimentin (VIM), a component of intermediate filaments, is abundantly expressed in mesenchymal cells [62] and exerts inhibitory effects on E-cadherin expression, and cell–cell adhesion, while promoting cell migration and invasion [63]. Therefore, Vimentin is not only an EMT marker but also directly promotes EMT in EOC.

In EOC, EMT is induced by several signaling pathways, including transforming growth factor- β (TGF-β)/Smads, Wnt/β-catenin, PI3K/AKT, Hedgehog, Sonic, and Notch [64]. Wnt signaling promotes the localization of β-catenin into the nucleus, which, in turn, interacts with T-cell factors (TCF/LEF) to regulate transcription [65]. The pathway inhibits E-cadherin by promoting the expression of E-cadherin repressors, such as Snail, Slug, and TWIST [65,66]. TGF-β also enhances EMT through its downstream mediators, SMAD2, SMAD3, and SMAD4 [67]. In addition, the MAPK and PI3K/AKT pathways, activated by many growth factors, or through cross-talks with other signaling molecules, also play critical roles in promoting EMT. For example, epidermal growth factor (EGF) signals through the ERK1/2 and PI3K/AKT pathways to induce EMT [68]. Hepatocyte growth factor (HGF) acts through its receptor, c-Met, and enhances EMT by activating multiple signaling pathways, including MAPK, Wnt/β-catenin, and PI3K/AKT [69,70,71]. Hedgehog glioma-associated oncogene1 (Shh-Gli1) positively regulates EMT via crosstalk with PI3K-AKT [72]. In addition to functions in mitotic progression, Aurora kinase A (AURKA) has been reported to regulate EOC cell migration and invasion in vitro and in vivo [73]. Treatments with AUKA inhibitors, such as alisertib, inhibited migration, adhesion, and EMT via the PI3K/AKT/mTOR- and Sirtuin-1-mediated pathways [73,74], suggesting a potential therapeutic advancement in controlling EOC dissemination. Finally, focal adhesion kinase (FAK) is an important component of various pro-metastatic signaling pathways which promote cancer metastasis, including cell motility [75], cell survival [76,77], invasion [78,79], and EMT [80]. Increased FAK levels are found in several cancers, including EOC [79,80]. In addition, FAK activation, which is determined by p-FAK, increases with tumor progression [80].

Actin filament dynamics are regulated strictly to maintain cell shape and control cell motility [53]. The increase in EOC cell mobility is mediated by actin filament remodeling via the activation of GTPase signaling pathways. For example, GTPase RAP1B has been reported to activate Src and JNK to facilitate integrin-mediated actin remodeling and thereby promote metastasis [81]. DAAM1, which is upregulated in EOC tumors, activates RHOA, induces the formation of microfilaments, and promotes cell migration and invasion [82]. In addition, Lim kinase 1 (LIMK1), a member of serine-threonine protein kinases that acts downstream of RHO GTPase signaling, also participates in actin remodeling in EOC [83]. LIMK1 is a key player in the reorganization of the actin cytoskeleton by inactivating actin-binding factor cofilin through phosphorylation [84]. LIMK1 protein levels are upregulated in EOC and correlated with poor differentiation [83]. In addition, knockout of LIMK1 inhibited migration and invasion of EOC cells [83], supporting its role in promoting EOC cell mobility.

Most EOC metastasis occurs in the peritoneal cavity. Once escaping the primary site, ovarian tumor cells transit in the ascitic fluid as single cells or aggregated cells, referred to as spheroids, and exhibit cancer stem-like properties [85,86]. Cancer cells then adhere to the mesothelium lining of the peritoneum through the binding of integrin receptors to the extracellular matrix (ECM) elements of the mesothelial cells [53]. The integrin-ECM interaction was suggested to activate integrin-linked kinase (ILK) through phosphorylation, promoting a phosphorylation cascade of a variety of ILK-intracellular substrates, including protein kinase B (PKB/AKT), glycogen synthase kinase-3 (GSK-3), and myosin light chain at focal adhesions, and promoting cell adhesion and invasion to the mesothelium [87]. In addition, ovarian tumor cells increase the production of proteolytic enzymes, such as matrix metalloproteases (MMPs), which recognize and degrade ECM elements, enhancing invasive behavior. MMPs play a role in EMT and they are also activated by genes and signaling pathways that induce EMT [88]. In EOC, it has been reported that knockdown of SNAI1 reduced MMP2 but upregulated its inhibitor, TIMP2, suggesting that Snail induces MMP activity [89]. Moreover, EOC cells avoid apoptosis while detaching from primary sites and circulating in ascites or transiting to a distant location by resisting anoikis, a programmed cell death which is activated to inhibit anchorage-independent growth or cell adhesion to an inappropriate matrix [90]. Among steps that occur in cancer metastasis, escaping apoptosis is critical in tumor development and metastasis [91].

Interaction between cancer cells and the tumor environment also plays a role in metastasis. Hypoxia is commonly observed in fast-growing tumors with an insufficient supply of oxygen. Under hypoxic conditions, the association of stabilized hypoxia-inducible factor (HIF)-1α and HIF-2α [92] with HIF-1β induces the expression of downstream target genes that are involved in cell invasion, and metastasis [93]. LOX, one of the target genes induced by HIF-1 complex, has been shown to cross-link collagen and provide a linear track for cell migration [94,95]. In addition, HIF-1 complex modulates the downregulation of DMN2, resulting in decreased endocytosis, an energy-consuming cellular process [94]. Hypoxia has also been reported to down-regulate BRCA1 expression via Retinoblastoma-associated protein E2F transcription factor and suppresses homologous recombination in hypoxic cancer cells, potentially increasing genomic instability [96,97]. Furthermore, the behaviors of metastatic EOC cells are influenced by secreted factors residing in ascites. Cytokine CXCL12 and hyaluronic acid in ascitic fluid have been demonstrated to interact with CXCR4 and CD44 receptors on EOC cell surface respectively, stimulating cell migration, angiogenesis, and localization to the peritoneal surface [53,98,99,100].

Lastly, the metastasis of EOC cells is enhanced by an immunosuppressive microenvironment. Tumor-infiltrating lymphocytes (TILs), such as T cells, B cells, macrophages, and natural killer cells, were also found to be present in ascites and pelvic peritoneal biopsies of advanced ovarian cancer patients [101]. Among them, tumor-associated macrophages (TAMs) play a role in the suppression of adaptive immunity. TAMs induced the imbalance of Treg/Th17 and promoted angiogenesis and metastasis via cross-talk with endothelial cells in EOC [102,103]. In addition, TIL-produced cytokines, such as IL-6, IL-10, ARG-1, and CCL-2, have been reported to promote tumor progression and metastasis, and are involved in immune subversion [104,105]. In addition, EOC cells promote immune evasion via downregulating tumor-associated surface ligands. MHC class I chain-related molecules A and B (MICA and MICB) are widely expressed on epithelial tumor cells and targeted by cytotoxic lymphocytes such as CD8+ T cells and natural killer (NK) cells [106]. Downregulation and internalization of MICA/B have been reported in EOC [106,107], allowing EOC malignant cells to escape immune surveillance.

## 4. Dysregulation of miRNA Expression in Ovarian Cancer

The expression of miRNAs is highly specific to cell types and developmental stages [108,109]. However, aberrant expression of miRNAs is commonly observed in EOC and associates with its progression [59]. Many miRNAs have been identified to be differentially expressed in EOC. These changes in miRNA abundance are often associated with alterations in cell migration, invasion, and metastasis, as listed in Table 1. Abnormal levels of miRNAs have been detected in tumor tissues, plasma, urine, and/or ascitic fluids. Such dysregulation can be attributed to alterations in DNA copy number, epigenetic regulation, and miRNA biogenesis.

### 4.1. Aberrant Expression of Metastasis-Associated miRNAs in EOC

In EOC tumors compared to normal ovarian tissues, upregulation of miR-181a [110], miR-616 [111] and miR-590-3p [112], and downregulation of miR-125b [113], miR-148a-3p [114], and miR-375 [115] levels have been reported. However, inconsistent findings have also been reported. For example, miR-124-3p, miR-148a-3p, miR-203a, and miR-375 were detected exhibiting differential expression in EOC specimens with both downregulation and upregulation [114,116,117,118,119]. Several other miRNAs listed in Table 1 have also been shown to be either up- or down-regulated in different studies. The reasons for such discrepancies are unclear; however, it may be related to sample size and/or heterogeneity within tumor samples or between different EOC subtypes. In addition, some of these studies did not specify the subtypes of tumors or only used a few tumor samples. Different EOC subtypes have unique origins and specific molecular features and, therefore, it is possible to exhibit different miRNA expression patterns. To date, only a few studies have examined miRNAs in different subtypes of EOC. Using microarray analyses to compare serous, endometrioid, and clear cell tumors with normal ovarian tissues, Iorio et al. (2007) found that while some miRNAs were commonly up- or down-regulated among different subtypes of EOCs, some miRNAs were dysregulated only in a specific subtype [120]. It has also been indicated that miR-510 expression was higher in LGSC and CCC subtypes but lower in HGSC compared to normal ovarian tissues [121]. Choosing proper controls is a challenging task in EOC research. Many researchers used normal ovarian or adjacent non-cancerous tissues as controls. However, it is now believed that the majority of EOCs are originated outside the ovary [3,122]. For example, HGSCs are thought to be derived from fallopian tube (FT) and ovarian surface epithelium while ECs and CCCs are originated from endometriosis [3]. The origin of MCs and LSGCs is still unclear, but they are frequently found to be associated with borderline tumors [3]. Therefore, comparison between tumor tissues and normal ovarian tissues may not provide accurate information about miRNA dysregulation. A recent study compared miRNA expression profiles between endometriosis and EOC tissues and suggested the potential role of miR-93, miR-325, and miR-492 in the malignant transformation of endometriosis to EOC [123]. Further investigation of miRNA expression in different subtypes in comparison with their tissues of origin would provide insights into their diagnostic and/or prognostic significance.

In addition to tumor tissues, the aberrant expression of miRNAs has also been detected in extracellular vesicles, especially exosomes, of EOC patients [124]. Interestingly, malignant cells have been reported to secrete more exosomes when compared with normal cells [124]. Consistent with the dysregulation of miRNA levels found in EOC tissues, miR-590-3p [112] and miR-376a [125] were found to be upregulated in plasma and serum samples of EOC patients, respectively. Expressions of miR-200a [126] and miR-20a [106] were increased while miR-122 [127] and miR-199a [128] expressions were decreased in the serum of EOC patients. Furthermore, upregulation of miR-149-3p and miR-222-5p was detected in peritoneal exosomes which were isolated from ascites of EOC patients [129].

The dysregulation of miRNAs is correlated with EOC pathological features, such as tumor stage, grade, and lymph node and uterus invasion. Levels of miR-520h were gradually increased from stage I to stage III/IV of EOC tumors [130]. Upregulation of miR-520h was associated with increased ascite volumes and poor survival of EOC patients. In contrast, decreased levels of miR-26b were negatively correlated with tumor stage, grade, and ascite volumes [131,132]. In addition, abnormal miRNA levels have been reported to be associated with EOC metastasis. Bioinformatic analysis demonstrated that there was a correlation between downregulation of miR-216 with lymphovascular invasion, upregulation of miR-133a-2, miR-145, and miR-126 with uterus invasion, and upregulation of miR-302c with pelvic peritoneum invasion [120]. Furthermore, analysis from primary and metastatic EOC specimens indicated that downregulation of miR-124-2, miR-125b-1, miR-137, miR-203a, and miR-375 was highly associated with lymph node and distant metastasis [116]. miR-146a [133] and miR-19a [134] were also increased in metastatic EOC tumors comparing to the primary tumors, while miR-7 levels were decreased in metastatic EOC tumors compared to the primary tumors [68]. The upregulation and downregulation of these miRNAs are associated with advanced stage, lymph node metastasis, and survival of EOC patients. Therefore, miRNAs may potentially be used as prognostic biomarkers.

### 4.2. Dysregulations of miRNAs by Genetic and Epigenetic Alterations

Genetic alteration is one of the mechanisms underlying the dysregulation of miRNAs in cells. Using high-resolution array comparative genomic hybridization (aCGH), Zhang et al. revealed that 37.1% of genomic regions containing miRNA genes showed DNA copy number variations in EOC [255]. Among the miRNA genes analyzed, 21 out of 47 intronic miRNA genes showed high degrees of copy number variations. Further analyses showed that dysregulation of most of these miRNAs was associated with DNA copy number abnormalities [255]. Downregulation of let-7a3 was correlated with a loss of DNA copy number while upregulation of miR-9-1 and miR-213 was associated with a gain of DNA copy number [255]. Similarly, 3q26 amplification has been detected frequently in HGSC [143]. One of the miRNA precursors located in this region, *mir-551b*, is amplified in 1/3 of HGSC patients. The dominant form of its mature miRNA, miR-551b-3p, is also elevated in HGSC tissues compared to normal ovarian tissue and associated with poor prognosis. Furthermore, 32% of CCC patients showed 17q23-25 amplification which contains *mir-21* gene [256]. Although the correlation of 17q23-25 amplification and miR-21 overexpression is low, the presence of 17q23-25 amplification increased miR-21 overexpression in the CCC cases by 25%. These studies suggest that alteration of DNA copy number contributes to the dysregulation of some miRNAs.

Alteration in the methylation of CpG islands in miRNA gene promoters has also been linked to the dysregulation of miRNAs in EOC. Analysis of two datasets from GEO showed that miR-340 was downregulated in EOC tissues [207]. Interrogation of the 2-kb region upstream of *mir-340* by EMBOSS in EOC cells revealed three CpG islands. In addition, treatment with methylation inhibitors, 5-Aza-dC or TSA, increased the levels of miR-340 in EOC cells, suggesting that the downregulation of miR-340 is due to hypermethylation of its promoter [207]. Hypermethylation in the CpG islands of multiple miRNA genes, including *mir-124a-2*, *mir-127*, *mir-125b-1*, *mir-129-2*, *mir-137*, *mir-193a*, *mir-203a*, *mir-34b/c*, *mir-130b*, *mir-1258*, *mir-9-1*, *mir-9-3*, *mir-107*, *mir-130b*, *mir-124-3*, *mir-339*, and *mir-375*, has also been found in EOC tumors compared to the paired normal ovarian tissues [116,257,258]. A strong correlation between hypermethylation and downregulation in miR-125b-5p, miR-129-5p, miR-132-3p, miR-137, and miR-193a-5p was also observed [116]. Interestingly, hypermethylation of *mir-125b-1*, *mir-137*, *mir-34b/c*, *mir-203a*, *mir-130b*, and *mir-375* is associated with metastasis [116,257]. Similarly, hypomethylation in *mir-21*, *mir-203*, *mir-205*, and *mir-191* promoters has been reported in EOC tissues with the association with their overexpression [116,120]. Thus, hypomethylation and hypermethylation of the promoter regions of miRNA genes play an important role in the aberrant expression of miRNAs.

### 4.3. Dysregulation of miRNAs by Alteration in miRNA Biogenesis

The first step of miRNA biogenesis is the transcription of the pri-miRNAs. As a consequence, dysregulation of factors that regulate transcription in general may lead to a change in the abundance of mature miRNA. p53, whose mutations occur in 96% of cases of HGSC [259], promotes the expression of miR-145 and miR-34b/c, and mutations of p53 are associated with lower expression of these miRNAs [161,259,260]. In addition, it has been demonstrated that c-Myb binds directly to the promoter of *mir-520h* to induce its expression [130]. c-Myb has been reported to be upregulated in EOC tissues compared to aged-matched control tissues and activation of TGF-β signaling induced upregulation of c-Myb and miR-520h [130,261]. Similarly, PPARγ response elements (PPRE) have been identified in the *mir-125b* promoter and activation of PPARγ leads to an increase in miR-125b levels [262]. However, miR-125b is reported to be downregulated [262] while PPARγ is upregulated [263] in EOC tissues compared to normal ovarian tissues. Despite of the upregulation, PPARγ was shown to inhibit proliferation and induce apoptosis by modulating p63 and p73 upregulation [264]. Therefore, it is unclear if PPARγ contributes to the downregulation of miR-125b. On the other hand, several transcription factors have been shown to inhibit miRNA expression. Overexpression of an ETS transcription factor, PEA3, or its activation by EGF signaling, significantly reduced pri-miR-125a and mature miR-125a levels and the binding of PEA3 to the *mir-125a* promoter has been confirmed by ChIP analysis [236]. Similarly, miR-6089 transcription was found to be directly inhibited by c-Jun which is activated by MYH9 via the Wnt/β-catenin signaling pathway [233]. Aberrant activation of EGFR and Wnt/β-catenin signaling is well documented in EOC [66,265]. Therefore, it is possible that the hyperactivation of these signaling pathways contributes to the downregulation of miR-125a and miR-6089, respectively.

Once transcribed, pri-miRNAs are processed into pre-miRNAs and finally mature miRNAs. These processing events are critical rate-limiting steps that regulate the levels of mature miRNAs within cells [38]. Abnormalities in DNA copy number of *DICER1* and *AGO2* have been detected in 32.7% and 57.4% of primary EOC tumors, respectively [255]. In addition, single-nucleotide mutations were found in *DICER1* and *DROSHA* genes [266]; however, the functional roles of these mutations are unclear. Lower mRNA levels of DICER and DROSHA were detected in 60% and 51%, respectively, of EOC tumors examined and associated with advanced tumor stage and poor survival of EOC patients [266]. DDX1, an RNA-binding protein that was identified to associate with the microprocessor complex, has been reported to play a key role in miRNA processing [21,267,268]. Low DDX1 expression is significantly associated with poor overall survival of serous EOC [269]. Silencing of DDX1 reduced the levels of mature miR-200a, miR-29c, miR-141, and miR-101 [269]. Interestingly, phosphorylation of DDX1 by the protein kinase Ataxia telangiectasia mutated (ATM) in response to DNA damage enhanced pri-miRNA processing [269]. Dicer, DROSHA, AGO2, and cofactors play critical roles in the controlled expression of mature miRNAs, and their dysregulation can lead to abnormal miRNA levels. Further investigation on the dysregulation of these components and their effects on specific miRNA expression levels would provide an insight into the mechanisms of miRNA dysregulation in EOC.

Single-nucleotide polymorphisms found in miRNAs, referred to as miRSNPs, may also contribute to the alteration of mature miRNA levels. The genetic variant rs11614913 of miR-196a-2 has been detected in multiple cancer types, including gastric [270], head and neck cancer [271], and breast cancer [272]. Genotype distribution analysis of miR-196a-2 polymorphism revealed that CT and CC genotypes are frequently observed in EOC patients [273]. In addition, the recessive genetic model indicated that the risk of EOC is higher in CC genotype carriers compared to the ones carrying wild-type homozygous TT and CT alleles. Furthermore, it was shown that miR-196a-2 rs11614913 influenced the production of mature miR-196a-2. There was a significant upregulation of mature miR-196a-2 detected in the CC genotype compared to the TT genotype of EOC tissue samples [273]. However, a recent study showed no significant difference in the frequency of miR-196a-2 rs11614913 in HGSC patients compared to healthy controls [274]. Therefore, whether miR-196a-2 rs11614913 variant is a risk factor in EOC requires further study.

## 5. Roles of miRNAs in Ovarian Cancer Metastasis

**Figure 2:**
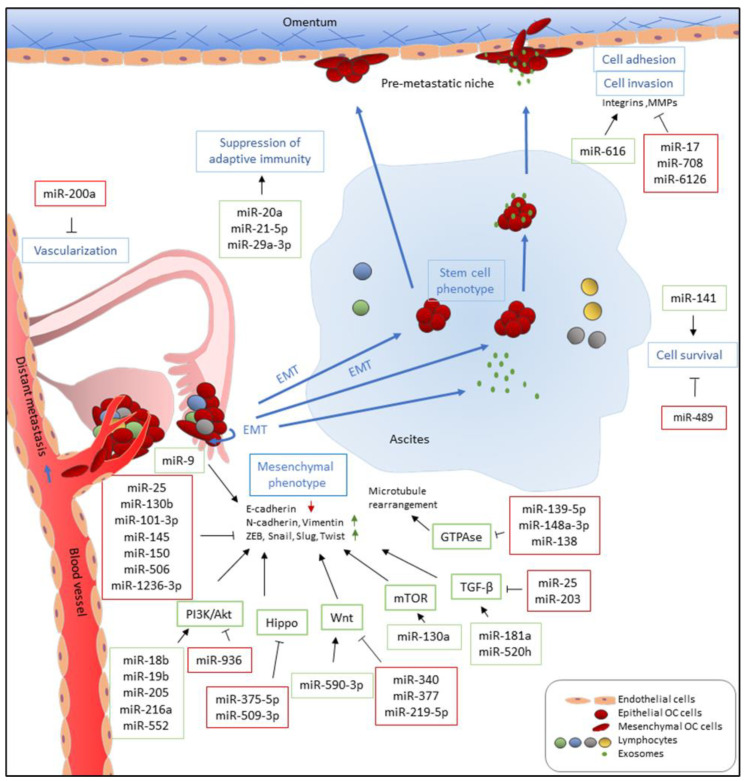
Functions of miRNAs in EOC metastasis. miRNAs which are upregulated and promote metastasis-related processes are depicted in green boxes while downregulated miRNAs which inhibit metastatic-related processes are listed in red boxes. miRNAs directly and indirectly regulate epithelial-to-mesenchymal transition (EMT) by targeting E-cadherin and E-cadherin repressors, such as ZEB, Snail, Slug, and Twist, and associated signaling pathways. miR-9 directly targets E-cadherin to activate EMT while miR-25, miR-101-3p, miR-130b, miR-145, miR-150, and miR-1236-3b directly inhibit expression of E-cadherin repressors, including *ZEB1*, *SNAI2*, and *TWIST1*. Multiple miRNAs, such as miR-18b, miR-19b, miR-205, miR-216b, and miR-552, promote EMT by targeting *PTEN*, leading to the activation of the PI3K/AKT pathway. In addition, upregulation of miR-590-3p, miR-130a, miR-181a, and miR-520h induces Wnt, mTOR, and TGF-β signaling, respectively, which are known pathways that promote EMT. In contrast, miR-936, miR-375-5p, miR-509-3p, miR-340, miR-377, miR-219-5p, miR-25, and miR-203 attenuate PI3K/AKT, Hippo, Wnt, and TGF-β signaling pathways and inhibit cell migration, invasion, and EMT. Furthermore, downregulation of miR-139-5p, miR-148a-3p, and miR-138, which has been reported to inhibit *ROCK*s and *LIMK1* expression, increases cell motility via GTPase signaling. Metastatic EOC cells float in ascites as single cells or spheroids which exhibit stem cell-like properties. To survive after detaching from primary site and inside ascites, metastatic EOC cells upregulate miR-141 and downregulate miR-489 to modulate anoikis resistance. In the transcoelomic pathway, EOC metastatic cells then adhere to the mesothelium lining and invade peritoneal organs. Upregulation of miR-616 and downregulation of miR-17 and miR-6126 increase cell adhesion via increased expression and activities of integrins and MMPs which recognize and degrade the extracellular matrix (ECM) of the mesothelial cells, respectively. In addition, downregulation of miR-708 increases focal adhesion formation through promoting focal adhesion kinase (FAK) activities. EOC metastasis occurs in an immune-suppressive environment which are modulated by miRNAs. miR-21-5p and miR-29a-3p promote adaptive immune suppression via upregulation of tumor-associated macrophages (TAMs) and induction Treg/Th17 imbalance while miR-20a downregulates *MICA/B* to avoid recognition by cytotoxic T-cells. Furthermore, EOC tumors increase vascularization and angiogenesis via downregulation of miR-200a, implicating them in distant metastasis through a perfusion pathway.

Numerous studies have reported the functions of miRNAs in EMT, cell migration, invasion, and metastasis in EOC. In addition, miRNAs also participate in inducing angiogenesis and modulating tumor microenvironments [102,105,106], which contribute to tumor metastasis (Figure 2). The majority of studied miRNAs exert negative regulatory effects on metastasis, while some miRNAs serve as positive regulators of metastasis. In addition, some miRNAs have been reported to exhibit both pro-metastatic and anti-metastatic effects, probably depending on the genes they targeted under different cancer contexts.

### 5.1. MicroRNAs that Promote Metastasis

Multiple miRNAs have been reported to promote EOC metastasis by targeting CDH1 as well as its associated pathways, such as AKT, TGF-β, and Wnt/β-catenin, to induce EMT. *CDH1* is directly inhibited by miR-9, which was found to be upregulated in serous ovarian cancer tissues and cell lines [242]. Overexpression of miR-9 in EOC cells promoted migration and invasion, as well as upregulating the mesenchymal markers, N-cadherin and Vimentin [242]. Furthermore, overexpression of miR-181a enhanced, while inhibition of miR-181a reduced, EMT, migration, invasion, and chemoresistance in vitro by targeting *SMAD7*, which negatively regulates TGF-β signaling [110]. Injection of miR-181a-overexpressing cells into the ovarian bursa of nude mice resulted in a significant increase in tumor burden and dissemination. These results, together with the findings that miR-181a is enriched in recurrent tumors compared to primary tumors and negatively correlated with patient survival [110], strongly suggest that miR-181a is a key regulator of EOC metastasis. Similarly, miR-520h targets *SMAD7*, resulting in EMT and tumor growth promotion, as supported by in vitro and in vivo studies [130]. Meanwhile, miR-182 inhibits BRCA1, a tumor suppressor involved in DNA repair, and MTSS1, a metastasis suppressor [275], and upregulates the EMT promoter HMGA2, thus increasing EOC cell invasiveness in vitro and tumor metastasis in vivo [136]. miR-182 was also found to target a transcription factor FOXF2 and to exert promoting effects on EOC cell migration and invasion [148].

PTEN, a tumor suppressor that negatively regulates PI3K/AKT pathway [276], is targeted by several miRNAs that promote metastasis. Specifically, miR-18b [144], miR-19b [145], miR-205 [153], miR-216a [154], and miR-552 [155] have been reported to directly target *PTEN* and enhance cell migration, invasion, and/or EMT in vitro when these miRNAs are overexpressed, but reduced these properties when endogenous miRNAs were inhibited. In vivo, miR-205 overexpression resulted in the formation of a larger number of intraperitoneal tumors [153]. Other target genes have also been identified for miR-205. In the EC subtype, miR-205 is upregulated in tumors and its overexpression enhances cell migration and invasion by targeting *ESRRG* [141]. The effect of miR-205 on promoting EOC cell migration and invasion has also been confirmed by other studies [277,278]. miR-130a was discovered to target *TSC1*, a negative regulator of the mTOR pathway [279], and increased proliferation, invasion, and EMT in vitro, as well as tumor growth and metastasis in vivo [138]. Interestingly, miR-130a is upregulated in HGSC, and, in response to inflammatory stimuli, NFκB binds to the miR-130a promoter to induce its expression [138]. These findings suggest that a mechanism by which inflammation promotes EOC metastasis is to induce miR-130a expression, which, in turn, activates mTOR signaling.

Studies from our lab have shown that miR-590-3p is upregulated in EOC tumor tissues and plasma samples [112]. Overexpression of miR-590-3p promotes, while inhibition of miR-590-3p suppresses, EOC cell proliferation, migration, and invasion, as well as spheroid formation in 3D cultures in vitro [112,159]. Subcutaneous and intraperitoneal injection of EOC cells overexpressing miR-590-3p revealed that miR-590-3p promotes tumor growth and metastasis [112]. We further showed that *FOXA2* is a direct target of miR-590-3p, while Versican (VCAN), a proteoglycan commonly overexpressed in cancer, is transcriptionally inhibited by FOXA2. Interestingly, we found that VCAN mRNA levels are positively correlated with vascular and lymphovascular invasion, and that low FOXA2/high VCAN is associated with poor patient survival [112]. In addition to FOXA2, miR-590-3p also targets cyclin G2 (*CCNG2*) and *FOXO3*. CCNG2 is an atypical cyclin that negatively regulates cell proliferation [280], migration, invasion, EMT, and tumor metastasis by suppressing Wnt/β-catenin [281]. FOXO3 has been shown to activate *CCNG2* transcription in EOC cells [282]. By targeting these two genes, miR-590-3p enhances β-catenin signaling [159]. These findings support the role of miR-590-3p in promoting EOC metastasis.

Matrix metalloproteinases (MMPs) play an important role in cell invasion and metastasis, and their activity is inhibited by tissue inhibitors of MMPs (TIMPs) [283]. It has been reported that miR-616 expression is positively correlated with EOC metastasis, advanced stage, poor differentiation, and lower patient survival, and its upregulation promotes invasion, migration, and EMT in vitro. *TIMP2* was identified as a direct target of miR-616. In addition, more lung metastatic nodules were observed when EOC cells transfected with miR-616 were injected through the tail vein into nude mice, suggesting that miR-616 promotes tumor metastasis [111].

Dicer, a key enzyme involved in miRNA biogenesis, was reported to be negatively correlated with advanced EOC stages and positively associated with better patient outcomes [266]. Rupaimoole et al. reported that *DICER1* was targeted by miR-630 in EOC under hypoxic conditions [156]. In vitro studies showed that miR-630 induced migration and invasion, as well as EMT. In an orthotopic mouse model of ovarian cancer, delivery of miR-630 via liposomal nanoparticles significantly enhanced tumor growth and distant metastasis [156]. Interestingly, treatment with a combination of anti-miR-630 and anti-vascular endothelial growth factor (VEGF) antibody resulted in a strong reduction in tumor growth and metastasis [156], suggesting the therapeutic potential of this approach. Paradoxically, another study found that Dicer was overexpressed in ovarian tumor stromal cells and played a pro-inflammatory and pro-metastatic role by down-regulating miR-6780b, which inhibits the NFκB pathway in fibroblasts [284]. Thus, Dicer may exert cell type-specific effects on EOC metastasis.

Thrombocytosis was reported to be positively associated with advanced stage and grade of EOC. In addition, it is frequently observed in patients with lymph node metastases, increased volumes of ascites, and decreased overall survival [158,285]. Tang et al. discovered that when EOC cells were cocultured with platelet microparticles (PMP), there was an increase in cell proliferation, migration, and EMT, suggesting the induction of EMT from platelet-delivered molecules [158]. In addition, in vitro analysis showed that PMPs contained miR-939 which promoted EMT in EOC cells. Finally, the uptake of miRNA-containing PMPs by EOC cells was suggested to be mediated by secretory phospholipase A2 type IIA [158]. These studies showed a mechanistic link between thrombocytosis and EOC progression. However, how miR-939 induces EMT remains to be investigated.

The immune microenvironment is known to be involved in EOC progression, including metastasis. miRNAs may also play a role in promoting EOC metastasis by altering the immune microenvironment. miR-29a-3p and miR-21-5p increased the ratio of regulatory and helper 17 T cells (Treg/Th17) and tumor growth and metastasis in vivo [102]. A higher Treg/Th17 ratio was observed in EOC and in metastatic peritoneal tissues. Both miRNAs were present at high levels in exosomes released from M2 macrophages. STAT3, which is involved in immune cell differentiation, was identified to be inhibited by both miRNAs [102]. These findings suggest that miR-29a-3p and miR-21-5p induce an immunosuppressive microenvironment to facilitate EOC development in part by targeting *STAT3*. Unlike in immune cells, STAT3 exerts tumor-promoting effects on EOC cells [286]. Interestingly, miR-551-3b may bind directly to *STAT3* promoter, leading to the recruitment of RNA polymerase II and transcription factor TWIST1, activating STAT3 transcription and promoting metastasis [143]. In addition, miR-551-3b promotes EOC stem cell proliferation, invasion, and chemoresistance by downregulating FOXO3 and TRIM31 [142]. Another instance of immune suppression is miR-20a, which may reduce natural killer cell cytotoxicity towards EOC cells by inhibiting MICA/B, resulting in immune evasion [106]. Serum levels of miR-20a were elevated in EOC subjects, with high expression associated with poor survival [106]. These studies provided insights into the role of miRNAs in cancer immune modulation.

### 5.2. MicroRNAs that Suppress Metastasis

#### 5.2.1. MicroRNAs Suppress Metastasis by Directly Targeting Transcription Factors that Regulate EMT Markers

Master regulators of EMT, including ZEBs, SNAIs, and TWISTs, operate largely as transcription repressors to repress CDH1 transcription [287]. Overexpression of miR-101-3p, miR-130b, and miR-1236-3p inhibits EOC cell invasion and migration, increases E-cadherin, and decreases mesenchymal markers by directly targeting *ZEB1* [165,168,178]. Similarly, *SNAI1* and *SNAI2* are targets of multiple miRNAs [119,167,175,222]. Using the TCGA database and tissue samples from EOC patients, Yang et al. identified miR-506 as a positive prognostic predictor of EOC patients [222]. MiR-506 was found to upregulate E-cadherin and downregulate SNAI2 and VIM, as well as inhibited cell invasion and migration. In addition to *SNAI2*, miR-506 also directly targeted *VIM* and *CDH2* [63]. Furthermore, in vivo miR-506 delivery via nanoparticles reduced the number and weight of tumor nodules in the intraperitoneal metastasis model [222], providing strong evidence that miR-506 suppresses metastasis. On the other hand, miR-145 has been shown to interact with the 3′ UTR of *TWIST1* and *SOX9* and exert inhibitory effects on EMT [193]. Importantly, when nude mice were inoculated intraperitoneally with miR-145-overexpressing EOC cells, there were fewer metastases and less ascites than the control mice [162]. These findings suggest that miR-145 suppresses metastasis and one of the possible mechanisms is to inhibit *TWIST1* and, therefore, EMT. miR-25 has also been reported to target *SNAI2* and inhibit TGF-β-induced EMT, cell migration, and invasion [167]. Through direct inhibition of EMT marker genes, these miRNAs suppress EMT and metastasis.

#### 5.2.2. MicroRNAs Suppress Metastasis by Targeting Growth Factors and Related Signaling Pathways

Several miRNAs have been shown to modulate the metastatic potential of EOC cells by targeting growth factors and their downstream signaling pathways. Ectopic expression of miR-139, miR-212, and miR-936 inhibited cell proliferation, invasion, and migration by targeting hepatoma-derived growth factor (*HDGF*), heparin-binding epidermal growth factor (*HBEGF*), and fibroblast growth factor 2 (*FGF2*), respectively [186,231,239]. Moreover, Li et al. found that low miR-936 expression in EOC tissues was associated with large tumor size, advanced stage, and lymphatic metastasis. They also demonstrated that miR-936 inhibited tumor growth in vivo, as well as significantly deactivated the PI3K/AKT pathway shown by decreased p-PI3K and p-AKT levels [231]. Similarly, miR-217 directly targeted insulin-like growth factor 1 receptor (*IGF1R*) and suppressed EOC cell proliferation, colony formation, invasion, and migration, as well as inhibited subcutaneous tumor growth [200]. In addition, miR-421 directly targeted platelet derived growth factor receptor A (*PDGFRA*) and inhibited CD44+/CD133+ serous human ovarian cancer stem cell proliferation, invasion, angiogenesis, and tumor growth [216]. These findings suggest that miR-421 exerts an anti-metastatic effect by suppressing PDGF signaling. Finally, miR-503-5p targets *CD97*, a member of the epidermal growth factor-seven transmembrane (EGF-TM7) family that induces JAK2/STAT3 signaling [288,289], to inhibit EOC cell proliferation, migration, and invasion [221].

Several anti-metastatic miRNAs have been reported to negatively regulate the Wnt signaling in EOC. Using EOC tissue samples and the TCGA database, Huang et al. and Yu et al. identified miR-340 and miR-377 as positive prognostic biomarkers of EOC patients [207,213]. By targeting Four-and-a-half LIM domains protein 2 (*FHL2*) and Cullin 4A (*CUL4A*), miR-340 and miR-377 decreased EOC cell migration and invasion, downregulated the Wnt/β-catenin pathway, and inhibited EMT [207,213]. Furthermore, miR-340-overexpressing EOC cells decreased the volume of ascites and peritoneal metastases in vivo. In addition to *FHL2*, miR-340 also targets *NFKB1* [208]. These findings strongly support a role for miR-340 in inhibiting EOC metastasis. miR-219-5p also inhibited cell proliferation, invasion, and migration, and downregulated the Wnt/β-catenin pathway by targeting *TWIST1* [202]. Overexpression of miR-219-5p downregulated both TWIST and β-catenin levels [202], suggesting that decreased expression of TWIST led to the downregulation of β-catenin via AKT/GSK3β axis [290]. Besides modulating TWIST expression directly, miR-219-5p was reported to target the oncofetal protein, high-mobility group A2 (*HMGA2*), and reduced subcutaneous tumor growth in vivo [203]. These findings show that these miRNAs have anti-metastatic effects and one of the mechanisms is by downregulating the Wnt/β-catenin signaling pathway. Additional studies using in vivo EOC metastasis models are required to further confirm their roles in suppressing EOC metastasis.

Yes-associated protein 1 (YAP1) is a downstream target of the Hippo signaling pathway which promotes the progression of various tumors, including colorectal, bladder, and liver cancers [291,292,293]. Several miRNAs have been reported to target *YAP1* in EOC. miR-509-3p, which was identified as a positive prognostic predictor in EOC patients, inhibited EOC cell invasion, migration, and spheroid formation by targeting *YAP1* [164], suggesting a potential role in inhibiting EOC metastasis. Similarly, miR-375-5p also directly targeted *YAP1* and inhibited EOC cell proliferation, invasion, migration, and EMT. Importantly, live animal bioluminescence imaging revealed that inhibition of miR-375-5p resulted in an increase in primary tumor growth and liver and spleen metastases [115], supporting a role for miR-375-5p in repressing EOC metastasis.

Regulation of the Rho pathway by miRNAs modulates cell motility and tumor metastasis [294]. Chen et al. reported that miR-106b, whose expression in EOC tissues was negatively associated with tumor grade and stage, directly targeted *RHOC* and reduced cell proliferation, invasion, and migration, as well as inhibited tumor growth in vivo [171]. Similarly, Rho-associated coiled-coil-containing protein kinases (ROCKs), including ROCK1 and ROCK2, are targets of miR-148a-3p and miR-139-5p, respectively [114,188,189]. Overexpression of miR-148a-3p and miR-139-5p inhibited cell proliferation, invasion, and migration in vitro, as well as suppressed EOC growth in nude mice [114,188,189]. On the other hand, miR-138 targets *LIMK1*, which is activated by signaling through the Rho family GTPases and reported to be a marker for malignant progression in EOC, to inhibit EOC cell proliferation, invasion, and migration [83].

#### 5.2.3. MicroRNAs Suppress Metastasis by Regulating Adhesion Molecules

Several miRNAs have been reported to downregulate FAK signaling to inhibit EOC metastasis. miR-708, a significantly downregulated miRNA in highly invasive EOC tissues with advanced stages and metastasis, was shown to inhibit focal adhesion formation, cell invasion, and migration, accompanied by decreased p-FAK and p-Paxillin expression, by targeting Ras family small GTP-binding protein (*RAP1B*) [81]. Overexpression of miR-708 reduced abdominal metastases in the orthotopic metastasis model [81], providing strong evidence that miR-708 suppresses EOC metastasis. In addition, miR-4454 inhibited cell invasion and migration by targeting secreted protein acidic and rich in cysteine (*SPARC*), a glycoprotein associated with metastatic EOC [295], to inhibit FAK activity. Importantly, when miR-4454-overexpressing EOC cells were inoculated intraperitoneally to the nude mice, fewer peritoneal metastases were observed [232]. These findings indicate that miR-4454 inhibits EOC metastasis and one possible mechanism is to suppress FAK activation.

The integrin α5β1 pair is a negative prognostic biomarker in EOC patients and participates in cancer cell adherence and mesothelial layer clearance in EOC metastasis [296,297,298]. miR-17, whose expression was negatively correlated with *ITGA5* and *ITGB1* expression in various EOC cell lines, decreased cell adhesion and invasion, as well as reduced peritoneal metastatic nodules in vivo by targeting *ITGA5* and *ITGB1*. In addition, miR-17 also repressed ILK phosphorylation and MMP-2 expression [170]. miR-6126, secreted by EOC cells via exosomes, acts on endothelial cells to suppress angiogenesis and on EOC cells to inhibit migration and invasion by directly targeting *ITGB1* [234]. Interestingly, miR-6126 delivery via DOPC nanoliposomes into an orthotopic mouse model of ovarian cancer suppressed tumor growth and angiogenesis. Moreover, miR-6126 was found to correlate with better overall survival of EOC patients [234]. These findings strongly support that miR-6126 suppresses metastasis.

#### 5.2.4. MicroRNAs Suppress Metastasis by Directly Targeting HOX Genes

Homeobox (HOX) genes encode developmental regulators which are crucial for growth and differentiation. The dysregulation of HOX genes has been observed in various cancers [299]. HOXA10 is overexpressed in EOC and is associated with poor survival [300]. By targeting *HOXA10* and *CCR2*, miR-135a, which is a positive prognostic biomarker in EOC patients, decreased EOC cell proliferation, adhesion, migration, and invasion, reduced Bcl-2 and N-cad expression, as well as increased p53, caspase-3, and E-cadherin expression. Importantly, overexpression of miR-135a reduced subcutaneous tumor growth and lung metastases [182,183], suggesting that miR-135a suppresses metastasis. Similarly, miR-665 also targets *HOXA10* and inhibits EOC cell proliferation and migration [229]. On the other hand, miR-612 and miR-202-5p directly target *HOXA13* and *HOXB2*, respectively. Both miRNAs were found to inhibit cell proliferation, invasion, and migration [196,227]. In addition, miR-202-5p inhibited EMT in vitro and was identified as a positive prognostic predictor in EOC patients [196].

#### 5.2.5. MicroRNAs Suppress Metastasis by Directly Targeting HIF

EOC cells with high levels of hypoxia-inducible factor (HIF) are more likely to disseminate from primary sites to the peritoneal cavity [301]. Moreover, HIF enhances tissue remodeling genes and is associated with EOC patient morbidity and mortality [302]. Joshi et al. reported that HIF transcriptionally suppressed miR-199a-5p in EOC cells, and miR-199a-5p interacted with the 3′ UTR of *HIF1A* and *HIF2A* to downregulate their levels, forming a feedback loop [94]. Ectopic miR-199a-5p expression decreased cell migration in vitro and inhibited tumor growth and peritoneal seeding upon intraperitoneal injection of EOC cells in vivo. The down-regulation of HIF also resulted in a decrease in LOX, an ECM remodeling enzyme and a negative prognostic biomarker in EOC patients [94]. LOX has also been reported to promote EOC cell migration and metastasis [95]. These findings provide strong evidence that miR-199a-5p suppresses metastasis, and its indirect regulation of the matrix/tissue remodeling gene expression may be one of the underlying mechanisms.

### 5.3. miRNAs that Have Been Reported to Promote and Inhibit Metastasis

Several miRNAs have been found to exert differential effects on metastasis. For example, the miR-200 family (miR-141/200a/200b/200c/429) has been reported to be either upregulated or downregulated in aggressive EOC and both expression levels were associated with poor survival [120,126,222,248,249,251,303]. Several studies have reported tumor-promoting effects of miR-141 in EOC, including enhancing cell proliferation, survival, anoikis resistance, chemoresistance, and peritoneal metastasis [245,304]. Mechanistically, miR-141 has been reported to target *KLF12* and *SIK1*, which are known to exert tumor-suppressive functions, inhibiting anoikis and p53-dependent apoptosis in EOC [245,305]. However, another study found that miR-141 and miR-200a induced tumor growth in a mouse model, but also sensitized tumors to chemotherapeutic drugs by targeting p38α to regulate oxidative stress [246]. miR-141 [134,247] and miR-200a [134] have also been reported to have inhibitory effects on EOC cell migration and invasion; however, a direct target was not determined in these studies. Similarly, miR-200a was reported to promote cell migration and invasion by targeting *PTEN* in a HGSC cell line [3], OVCAR3 [248], while impeding vasculogenic mimicry through targeting *EPHA2*, leading to decreased cell invasion in SKOV3 cells [250], which are thought to represent the EC subtype [3]. Opposing to the association between the upregulation of miR-200a and miR-200b and poor clinical outcomes, miR-200a and miR-200b overexpression reduced IL-8 and CXCL1 levels and inhibited cell migration, invasion, angiogenesis, and intraperitoneal metastasis in vivo [249]. Interestingly, growing evidence indicates that the miR-200 family may inhibit EMT via direct repression of *ZEB1/2* [134,252,306]. Therefore, it has been proposed that the expression of the miR-200 family members is downregulated initially to enhance EMT and increase the invasiveness of EOC cells, and is then upregulated to induce mesenchymal-to-epithelial-transition (MET) and achieve re-epithelialization of tumor cells in distant metastatic sites [307]. It has been suggested that the downregulation of miR-200s occurs during the progression of tumor cells gaining metastatic capability [308]. Furthermore, metastatic EOC tissues showed increased MET markers when compared to primary cancer tissues [309], suggesting a possible downregulation of the miR-200 family at some point during the re-epithelialization process. Whether or not the miR-200 family has subtype- and/or stage-specific effects on metastasis requires further investigation.

In addition to the miR-200 family, other studies reported that in the SKOV3 cell line, miR-9 either increased migration and invasion by targeting *CDH1* or inhibited migration and invasion by targeting *TLN1* [242,244]. These studies also examined miR-9 expression in tissue samples, but observed a higher expression in serous EOC metastases compared to primary tumors [242] and lower expression in recurrent serous EOC tumors compared to primary EOC tissues [243]. The opposing effects of miR-9 also have been reported in other cancers, with varied targets and functional roles which are more likely dependent on tissue or cell type [310,311,312]. Similarly, miR-203 promoted OVCA429 and OVCA433 cell proliferation and migration in vitro, as well as tumor metastasis in vivo, by targeting pyruvate dehydrogenase B (*PDHB*) and the subsequent enhancement of glycolysis [118]. In contrast, ectopic miR-203 expression in SKOV3 and OVCAR3 cells attenuated EMT, cell migration, and invasion in vitro and tumor growth in vivo, by targeting *SNAI2* [118] and *BIRC5* [254]. The reasons for such discrepancies are not clear; however, the relative levels of different target genes and their functions in tumor development may contribute to the differential effects of miRNAs.

## 6. Conclusions and Future Direction

Cancer metastasis is one of the main factors that leads to poor clinical outcomes for EOC patients. Accumulating evidence demonstrates that miRNAs play important roles in EOC metastasis. Aberrant expression of miRNAs has been reported in EOC. Such dysregulation can be attributed to alterations at the DNA level, such as amplification and hypermethylation at the promoter regions of miRNA genes. In addition, altered transcriptional controls and defects in miRNA biogenesis machinery also contribute to the abnormality of miRNA levels. Many studies have reported that miRNA expression profiles correlate with clinical features, such as tumor stage, grade, and overall survival of patients, raising the possibility of using miRNAs as diagnostic and/or prognostic markers.

Due to the heterogeneity of EOC, it is a challenge to find effective biomarkers for detecting EOC in different tumors [313]. miRNA profiling studies have sometimes reported inconsistent findings. One of the underlying issues could be the controls used. Some researchers used normal ovarian tissues as the control while others used benign tumors. Methylation patterns of some miRNA genes have been shown to be correlated with metastasis [116,257]. In addition, miRNAs are detected in biological fluids, which can serve as a non-invasive tool for EOC diagnosis. Further efforts in validating the specificity and sensitivity of miRNA signatures in a large cohort of EOC patients are needed for the development of miRNAs as diagnostic and prognostic biomarkers.

Metastasis of ovarian cancer is orchestrated by several interconnected biological processes, including EMT, increase in cell mobility and migration, destruction of the ECM, formation of spheroids, avoidance of apoptosis, angiogenesis, and immune suppression [91]. Some miRNAs have been reported to promote metastasis, mainly by targeting negative regulators of these processes. On the other hand, most miRNAs that have been studied exert suppressive effects on metastasis, mainly by inhibiting transcription factors that induce the expression of mesenchymal markers, or key signaling pathways that promote EMT, motility, and tumor angiogenesis. Therefore, the upregulation of metastasis-promoting miRNAs and downregulation of metastasis-suppressing miRNAs would lead to a dysregulated signaling network and promote metastasis. However, more work is required to better understand the role of miRNAs and the underlying mechanisms by which they regulate metastasis. Among the studies reported so far, some are comprehensive, but most only examined the effects of miRNAs using established EOC cell lines in vitro. Therefore, further in vivo experiments would verify the roles of those miRNAs in metastasis of EOC. Moreover, miRNAs can target many genes and it is possible that they could exert tumor-promoting or tumor-suppressive effects, depending on the relative abundance and/or functions of target genes in different tumor contexts. Most studies have been focused on one or a few target genes. Additional studies in identifying critical targets that are directly involved in the induction of EOC metastasis would enhance our understanding of the roles of miRNAs in these processes. Finally, EOC consists of multiple histological subtypes, each one with unique origins and distinct molecular features [3]. More work on examining the dysregulation and functions of miRNAs in different subtypes of EOC is warranted as it may help to develop precise therapeutics. miRNAs have been suggested as promising therapeutic targets for cancer treatment [314]. miRNA-based therapies have been established for lung cancer treatment and further trials are anticipated to address clinical treatment efficacy [314]. It is possible that the restoration of down-regulated miRNAs that inhibit metastasis or inhibition of up-regulated miRNAs that promote metastasis could be used in the future as a therapeutic approach for EOC.

## Figures and Tables

**Table 1:** Metastasis-related miRNAs in epithelial ovarian cancer (EOC).

miRNA/ miRNA Family	Altered Expression in EOC and Clinical Significance	Target Gene	In Vitro/In Vivo Effects	Citations
**Pro-Metastatic miRNAs**				
miRNAs upregulated in tumor tissues				
miR-19a	Upregulated in metastatic HGSC tissues compared to normal ovarian tissues	ND	ND	[134]
miR-182	Upregulated in HGSC tissues compared to fallopian tube tissues	*MTSS1*	Promotes cell invasion in vitro, and tumor growth and metastasis in vivo	[135,136]
miR-328-3p	Upregulated in cancer stem-like cells isolated from HGSC tissues compared to bulk cancer cells	*DDB2*	Increases ALDH+ population and promotes spheroid formation and CSC properties in vitro and tumor growth and metastasis in vivo	[137]
miR-130a	Upregulated in HGSC tissues compared to normal fallopian tube tissues	*TSC1*	Promotes cell proliferation, invasion, and EMT and tumor growth and metastasis in vivo	[138]
miR-301b-3p	Upregulated in HGSC tissues compared to paired adjacent normal tissues; positively correlated with tumor stage, lymph node metastasis, and poor survival	*CPEB3*	Promotes cell migration and invasion in vitro	[139]
miR-520h	Upregulated in EOC tissues compared to benign ovarian tumors and highest in HGSC compared to MC, EC, and CCC subtypes; correlated with tumor stage, increased ascites, lymph node metastasis, and poor survival	*SMAD7*	Promotes cell proliferation, invasion, and EMT in vitro and tumor growth and metastasis in vivo	[130]
miR-21	Upregulated in serous EOC, EC, and MC tissues compared to ovarian cysts and normal ovarian tissues; positively correlated with tumor stage and lymph node metastasis	ND	Promotes cell proliferation, migration, and invasion in vitro	[140]
miR-205	Upregulated in EC subtype compared to normal endometrial tissues	*ESRRG*	Promotes cell proliferation, migration, and invasion in vitro	[141]
miR-146a	Upregulated in omental metastatic serous EOC tumors compared to primary EOC tumors	ND	Promotes spheroid formation and cisplatin resistance in vitro	[133]
miR-551b	Upregulated in recurrent serous EOC tissues compared to primary EOC tumors; associated with advanced stage	*FOXO3* *TRIM31*	Promotes cell proliferation, invasion, and colony formation in vitro and tumor burden in vivo	[142]
miR-551b-3p	Upregulated in HGSC tissues compared to normal ovarian tissues; associated with poor outcome	*STAT3* promoter	Promotes cell proliferation, spheroid formation, and survival in vitro and tumor burden in vivo	[143]
miR-18b	Upregulated in EOC tissues compared to normal ovarian tissues; positively correlated with tumor grade and lymph node metastasis	*PTEN*	Promotes cell migration and invasion in vitro	[144]
miR-19b	Upregulated in EOC tissues compared to matched non-tumor tissues; positively correlated with tumor stage and lymph node metastasis	*PTEN*	Promotes cell migration and invasion in vitro	[145]
miR-23a	Upregulated in EOC tissues compared to adjacent normal tissues	*DLG2*	Promotes cell proliferation, migration, and invasion in vitro and tumor growth in vivo	[146]
miR-181a	Upregulated in recurrent EOC tissues compared to primary EOC tissues; associated with poor survival	*SMAD7*	Promotes cell migration, invasion, survival, and EMT in vitro and tumor growth and metastasis in vivo	[110]
miR-181b	Upregulated in EOC tissues compared to normal ovarian tissues	*LATS2*	Promotes cell proliferation and invasion in vitro	[147]
miR-182-5p	Upregulated in EOC tissues compared to non-tumor ovarian tissues	*FOXF2*	Promotes cell proliferation, and invasion in vitro and tumor growth in vivo	[148]
miR-194	Upregulated in EOC tissues compared to normal ovarian epithelium tissues	*PTPN12*	Promotes cell proliferation, migration and invasion in vitro	[149]
miR-196a	Upregulated in EOC compared to paired normal ovarian tissues; positively correlated with tumor stage, and lymph node metastasis	ND	ND	[150]
miR-205	Upregulated in EOC tissues compared to normal ovarian tissues; correlated with tumor stage and poor survival	*TCF21* *PTEN* *SMAD4*	Promotes cell proliferation, migration, and invasion in vitro and tumor growth and metastasis in vivo	[151,152,153]
miR-216a	Upregulated in EOC tissues compared to normal ovarian tissues; correlated with tumor stage, lymph node metastasis, and poor survival	*PTEN*	Promotes cell migration and invasion and EMT in vitro	[154]
miR-552	Upregulated in EOC tissues compared to paired non-tumor tissues; associated with metastasis, recurrence, and poor survival	*PTEN*	Promotes cell proliferation, migration, and invasion in vitro	[155]
miR-616	Upregulated in EOC tissues compared to adjacent non-tumor tissues; associated with metastasis, tumor stage and grade, and poor survival	*TIMP2*	Promotes cell migration, invasion, and EMT in vitro and metastasis in vivo	[111]
miR-630	Upregulated in EOC tissues with high levels of hypoxia compared to low levels of hypoxia; associated with poor survival	*DICER1*	Promotes cell migration, invasion, and EMT in vitro and tumor growth and metastasis in vivo	[156]
miR-939	Upregulated in EOC tissues compared to matched adjacent normal tissues	*APC2*	Promotes cell proliferation, colony formation, cell migration, invasion, and EMT in vitro	[157,158]
miRNAs upregulated in secreted exosomes and circulating body fluids				
miR-376a	Upregulated in EOC tissues compared to paired adjacent normal tissues and in blood samples of EOC patients compared to healthy controls; associated with advanced stages	*KLF15* *CASP8*	Promotes cell proliferation, migration, and invasion in vitro and tumor growth in vivo	[125]
miR-590-3p	Upregulated in EOC tissues compared to normal ovarian tissues, and in plasma of EOC patients compared to those with benign gynecologic disorders; correlated with tumor grade	*FOXA2* *FOXO3* *CCNG2*	Promotes colony and spheroid formation, cell migration, and invasion in vitro and tumor burden in vivo	[112,159]
miR-29-3p	Upregulated in exosomes secreted by M2 macrophages compared to those derived from THP-1 cells; associated with poor survival	*STAT3*	Promotes Tregs/Th17 imbalance in vitro and tumor growth and metastasis in vivo	[102]
miR-30a-5p	Upregulated in urine samples of serous EOC patients compared to healthy controls and higher in stage I/II compared to stage III/V; associated with lymphatic metastasis	ND	Promotes cell proliferation and migration in vitro	[160]
miR-149-3p	Upregulated in peritoneal exosomes of EOC patients compared to healthy controls; associated with poor survival	ND	ND	[129]
miR-222-5p	Upregulated in peritoneal exosomes of EOC patients compared to healthy controls; associated with poor survival	ND	ND	[129]
**Anti-Metastatic miRNAs**				
miRNAs down-regulated in tumor tissues				
miR-145	Downregulated in HGSC compared to normal FT tissues	*MTDH* *TWIST* *SOX9* *HMGA2*	Inhibits cell proliferation, invasion, migration, EMT, and spheroid formation in vitro and tumor growth and metastasis in vivo	[161,162,163]
miR-509-3p	Positively associated with survival in HGSC	*YAP1*	Inhibits cell invasion, migration, and spheroid formation in vitro	[164]
miR-1236-3p	Downregulated in HGSC tissues compared to normal FT tissues	*ZEB1*	Inhibits cell invasion, migration, and EMT in vitro	[165]
miR-574-3p	Decreased in EOC tissues compared to normal ovarian tissues, significantly lower in serous EOC tissues compared to non-serous EOC tissues; negatively associated with tumor stage	*EGFR*	Inhibits cell invasion and migration in vitro	[166]
miR-25	Downregulated in integrated mesenchymal EOC subtype compared to epithelial EOC subtype based on TCGA database	*SNAI2*	Inhibits cell invasion, migration, and EMT in vitro and tumor growth and metastasis in the orthotopic xenograft mouse model	[167]
miR-101	Decreased in integrated mesenchymal OC subtype compared to integrated epithelial OC subtype from TCGA database	*ZEB1* *FN1*	Inhibits cell invasion, migration, and EMT in vitro and tumor growth and intraperitoneal metastasis in vivo	[168,169]
miR-7	Downregulated in metastatic EOC tissues from omentum or peritoneum compared to primary EOC tissues; associated with metastasis	*EGFR*	Inhibits cell invasion, migration, and EMT in vitro	[68]
miR-17-5p	ND	*ITGA5* *ITGB1*	Suppresses cell adhesion and invasion in vitro and peritoneal metastasis in vivo	[170]
miR-106b	Decreased in EOC tissues compared to normal ovarian tissues and benign tumors; negatively associated with tumor stage and grade	*RHOC*	Inhibits cell proliferation, invasion, and migration in vitro and tumor growth in vivo	[171]
miR-23b	Decreased in EOC tissues compared to normal ovarian tissues and benign tumors	*CCNG1*	Inhibits cell proliferation, invasion, and migration in vitro and tumor growth in vivo	[172]
miR-26b	Downregulated in EOC tissues compared to normal ovarian surface epithelial tissues; inversely correlated with stage and grade, and higher risk with distant metastasis, recurrence, and poor survival	*KPNA2*	Inhibits cell proliferation, migration, spheroid formation, and EMT in vitro and tumor growth and lung metastasis in vivo	[131,132]
miR-29c-3p	Downregulated in EOC tissues compared to normal ovarian tissues	ND	Inhibits cell proliferation, invasion, migration, and EMT in vitro	[173]
miR-32	Downregulated in EOC tissues compared to adjacent normal tissues and in recurrent EOC tissues compared to primary tumors	*BTLA*	Inhibits cell proliferation, migration, and invasion in vitro	[174]
miR-34a	Downregulated in EOC tissues compared to paired adjacent normal ovarian tissues; negatively associated with late stage	*SNAI1*	Inhibits cell invasion, EMT, spheroid formation, and apoptosis in vitro	[175]
miR-100	Downregulated in EOC tissues compared to matched adjacent normal ovarian tissues; negatively associated with advanced stage, lymph node metastasis, and poor survival	*PLK1*	Inhibits cell proliferation in vitro	[176]
miR-124	Downregulated in EOC tissues compared to normal ovarian tissues, and lower in metastatic EOC tissues compared to primary EOC tissues	*SPHK1* *PDCD6*	Inhibits cell proliferation, colony formation, cell invasion, and migration in vitro	[117,177]
miR-130b	Downregulated in EOC tissues compared to adjacent non-tumor tissues	*ZEB1* *STAT3*	Inhibits cell invasion, migration, and EMT in vitro	[178]
miR-133a	Downregulated in EOC tissues compared to normal ovarian tissues; negatively associated with late stage and lymph node metastasis	ND	Inhibits cell proliferation, invasion, and migration and induces apoptosis in vitro	[179]
miR-133a-3p	Downregulated in EOC tissues compared to adjacent non-tumor tissues	ND	Inhibits cell proliferation, invasion, and EMT in vitro	[180]
miR-133b	Downregulated in EOC tissues compared to normal ovarian epithelial tissues and benign ovarian cyst tissues; negatively associated with tumor grade and lymph node metastasis	*CTGF*	Inhibits cell invasion, migration, and EMT in vitro	[181]
miR-135a	Downregulated in EOC tissues compared to ovarian cystadenomas; negatively associated with stage, lymph node metastasis, and poor survival	*HOXA10*	Inhibits cell proliferation and adhesion and promotes apoptosis in vitro	[182]
miR-135a-3p	Downregulated in EOC tumors compared to paired adjacent non-tumor tissues; negatively correlated with advanced stage and poor OS	*CCR2*	Inhibits cell proliferation, migration, invasion, and EMT in vitro and tumor growth and lung metastasis in vivo	[183]
miR-137	Downregulated in EOC tissues compared to paired adjacent tissues	*SNAI1*	Inhibits cell invasion, spheroid formation, and EMT in vitro	[175]
miR-138	Downregulated in EOC tissues compared to contralateral normal ovarian tissues; negatively associated with lymph node metastasis	*SOX4* *HIF1A* *SOX12* *LIMK1*	Inhibits cell proliferation and invasion in vitro and tumor metastasis in the orthotopic xenograft mouse model	[83,184,185]
miR-139	Downregulated in EOC tissues compared to paired adjacent normal tissues	*HDGF*	Inhibits cell proliferation, migration, and invasion in vitro	[186]
miR-139-3p	Downregulated in EOC compared to adjacent normal ovarian; negatively associated with advanced stage, lymph node metastasis, and poor survival	*ELAVL1*	Inhibits cell proliferation, colony formation, invasion, and migration in vitro and tumor growth and lung metastasis in vivo	[187]
miR-139-5p	Downregulated in EOC tissues compared to precancerous tissues; negatively associated with stage, lymph node metastasis and poor survival	*ROCK2*	Inhibits cell proliferation, colony formation, migration, and invasion in vitro and tumor growth in vivo	[188,189]
miR-145-5p	Downregulated in EOC tissues compared to paired adjacent normal ovarian tissues	*SMAD4*	Inhibits cell proliferation and migration and promotes apoptosis in vitro	[190]
miR-148a-3p	Downregulated in EOC tissues compared to adjacent non-tumor tissues	*ROCK1*	Inhibits cell proliferation, invasion, and migration in vitro, as well as tumor growth in vivo	[114]
miR-152	Downregulated in EOC tissues compared to paired adjacent normal ovarian tissues	*ADAM17* *WNT1* *ERBB3*	Inhibits cell proliferation, invasion, migration, and EMT in vitro	[178,191]
miR-150	Downregulated in EOC tissues compared to normal ovarian tissues; negatively correlated with advanced tumor stage and grade and poor survival	*ZEB1*	Inhibits cell proliferation, invasion, migration, EMT, and spheroid formation in vitro	[133,192]
miR-186	Downregulated in cisplatin-resistant EOC cells compared to cisplatin-sensitive EOC cells; decreased expression is associated poor OS	*TWIST1*	Inhibits cell proliferation, invasion, migration, and EMT in vitro and tumor growth and EMT in vivo	[193]
miR-193b	Downregulated in EOC compared to matched adjacent normal ovarian tissues and in omental metastasis compared to paired adjacent normal omentum; negatively correlated with stage, grade, ascites, lymph node metastasis, tumor size, and poor survival	*uPA*	Inhibits cell adhesion, proliferation, colony formation, invasion and migration in vitro, and inhibits tumor growth and metastasis in the orthotopic xenograft mouse model	[194,195]
miR-199a-5p	Downregulated in EOC cells under hypoxia compared to in normoxic condition	*HIF1A* *HIF2A*	Inhibits cell migration in vitro and inhibits tumor growth and peritoneal seeding in vivo	[94]
miR-202-5p	Downregulated in EOC tissues compared to paired adjacent normal ovarian tissues; positively associated with patient survival	*HOXB2*	Inhibits cell proliferation, invasion, migration, and EMT in vitro	[196]
miR-206	Downregulated in EOC tissues compared to noncancerous glioma tissues; negatively associated with lymph node and distant metastasis	*c-MET* *CCND1* *CCND2*	Suppresses cell proliferation, migration, and invasion in vitro	[197,198]
miR-208a-5p	Downregulated in metastatic EOC tissues compared to nonmetastatic EOC tissues	*DAAM1*	Inhibits cell invasion, migration, and microfilaments formation in vitro	[82]
miR-215	Downregulated in EOC tissues compared to adjacent normal; negatively associated with stage and lymph node metastasis	*NOB1*	Inhibits cell proliferation, colony formation, migration, and invasion in vitro and tumor growth in vivo.	[199]
miR-217	Downregulated in EOC tissues compared to paired adjacent normal ovarian tissues; negatively associated with stage, histological grade, and lymph node metastasis	*IGF1R* *IL6*	Inhibits cell proliferation, colony formation, invasion, and migration, and reduces M0 macrophages differentiation in vitro and tumor growth in vivo	[105,200]
miR-218	Downregulated in EOC tissues compared to adjacent normal; negatively associated with stage and lymph node metastasis	*RUNX2*	Inhibits cell proliferation, colony formation, invasion, and migration in vitro and tumor growth in vivo	[201]
miR-219-5p	Decreased in EOC tissues compared to adjacent normal tissues	*TWIST1* *HMGA2*	Inhibits cell proliferation, invasion, and migration in vitro and tumor growth in vivo	[202,203]
miR-335	Downregulated in EOC tissues compared to normal ovarian tissues, in omental metastatic tissues compared to primary tumors; negatively associated with poor survival and recurrence	ND	ND	[204]
miR-338-3p	Downregulated in EOC tissues compared to normal fallopian samples based on TCGA database; negatively associated with stage, grade, and metastasis	*MACC1* *RUNX2*	Inhibits cell proliferation, colony formation, invasion, migration, and EMT in vitro and tumor growth and metastasis in vivo	[70,205,206]
miR-340	Downregulated in EOC tissues compared to normal adjacent ovarian	*FHL2* *NFKB1*	Inhibits cell proliferation, invasion, and migration in vitro and tumor growth and intraperitoneal metastasis in vivo	[207,208]
miR-363	Downregulated in EOC compared to paired adjacent normal ovarian tissues; negatively associated with advanced stage, lymph node metastasis, and poor prognosis	*NOB1*	Inhibits cell proliferation, colony formation, invasion, and migration in vitro and tumor growth in vivo	[209]
miR-365	Downregulated in EOC tissues compared to adjacent normal ovarian tissues; negatively associated with stage, grade, and lymph node metastasis	*WNT5A*	Inhibits cell proliferation, colony formation, invasion, migration, and EMT in vitro, and tumor growth in vivo	[210]
miR-373	Downregulated in EOC tumors compared to benign epithelial ovarian tumors	*RAB22A*	Inhibits cell invasion, migration and EMT in vitro and peritoneal metastasis in vivo	[211,212]
miR-375	Downregulated EOC tissues compared to normal ovarian tissues	*YAP1*	Inhibits cell proliferation, invasion, migration, and EMT in vitro and tumor growth, metastasis, and EMT in vivo	[115]
miR-377	Downregulated in EOC tissues compared to adjacent normal ovarian tissues; positively correlated with survival	*CUL4A*	Suppresses cell proliferation, invasion, migration, and EMT in vitro	[213]
miR-494	Downregulated in EOC tissues compared to normal adjacent tissues; negatively associated with stage, tumor size, and lymph node metastasis	*SIRT1*	Inhibits cell proliferation, invasion, and migration in vitro	[214]
miR-378g	Downregulated in EOC tissues compared to normal ovarian tissues	*CHI3L1*	Inhibits cell proliferation, migration, and TGF-β1-induced EMT in vitro	[215]
miR-421	ND	*PDGFRA*	Inhibits cell proliferation, invasion, and tubule formation in vitro, and tumor growth and angiogenesis in vivo	[216]
miR-448	Downregulated in EOC tissues compared to adjacent normal ovarian tissues	*CXCL12*	Inhibits cell proliferation, migration and invasion in vitro and tumor growth in vivo	[99]
miR-450a	Downregulated in EOC tissues compared to normal ovarian tissues	*TIMMDC1* *MT-ND2* *ACO2* *ATP5B*	Inhibits cell clonogenicity, invasion, migration, and EMT and promotes anoikis in vitro and intraperitoneal tumor growth in vivo	[217]
miR-455	Downregulated in EOC tissues compared to normal adjacent tissues; negatively associated with stage, tumor size, and lymph node metastasis	*NOTCH1*	Inhibits cell proliferation and invasion in vitro	[218]
miR-489	Downregulated in EOC tissues compared to normal ovarian tissues; negatively associated with poor survival, stage, grade, lymph node, and distant metastasis	*XIAP*	Inhibits cell proliferation, invasion, migration and EMT in vitro	[219]
miR-497	Downregulated in EOC tissues compared to normal ovarian tissues; negatively associated with tumor stage, lymph node metastasis, and poor survival	*SMURF1*	Inhibition of cell migration and invasion in vitro	[220]
miR-503-5p	Downregulated in response to NF-kB pathway activation in SKOV3 cells	*CD97*	Inhibits colony formation, migration and invasion in vitro	[221]
miR-506	Decreased expression is associated with stage and poor survival in EOC	*SNAI2* *VIM* *CDH2*	Inhibits cell invasion, migration, and EMT in vitro, and EMT, tumor growth, and metastasis in the orthotopic xenograft mouse model	[63,222]
miR-532-5p	Downregulated in EOC compared to normal ovarian; negatively associated with stage, grade, and distant metastasis	*TWIST1*	Inhibits cell proliferation, colony formation, and invasion in vitro	[223]
miR-548c	Decreased in EOC tissues compared to normal ovarian tissues; negatively associated with advanced stage and poor survival	*TWIST1*	Inhibits cell proliferation, migration, invasion, stemness, and EMT in vitro	[224]
miR-584	Downregulated in EOC tissues compared to paracancerous tissues; negatively associated with distant and lymphatic metastasis and poor survival	*LPIN1*	Inhibits cell proliferation, colony formation, and migration in vitro	[225]
miR-596	ND	*LETM1*	Inhibits cell proliferation, invasion, and migration in vitro and tumor growth in vivo	[226]
miR-612	Downregulated in EOC tissues compared to matched non-tumor tissues	*HOXA13*	Inhibits cell proliferation, invasion, and migration, and promotes apoptosis in vitro	[227]
miR-654-3p	Downregulated in EOC cells compared to IOSE80 cells	*AKT3*	Inhibits cell invasion, migration and sphere formation in vitro and tumor growth in vivo	[228]
miR-665	Decreased in EOC tissues compared to normal ovarian tissues	*HOXA10*	Inhibits cell proliferation and migration in vitro	[229]
miR-708	Downregulated in EOC tissues compared to normal ovarian tissues; negatively associated with stage	*RAP1B*	Inhibits cell invasion, migration, cell adhesion, and EMT in vitro and tumor growth and metastasis in vivo	[81]
miR-874-3p/5p	Downregulated in EOC tissues compared to normal ovarian epithelial tissues	*SIK2*	Inhibits cell proliferation, colony formation, invasion, and migration in vitro	[230]
miR-936	Downregulated in EOC tissues compared to adjacent normal tissues; negatively associated with tumor size, stage, and lymphatic metastasis	*FGF2*	Inhibits cell proliferation, invasion, and migration in vitro and tumor growth in vivo.	[231]
miR-4454	Downregulated in metastatic EOC tissues compared to primary EOC tissues; positively associated with patient survival	*SPARC* *BAG5*	Inhibits cell proliferation, colony formation, migration, and invasion in vitro, and peritoneal metastasis in vivo.	[232]
miR-6089	Downregulated in EOC tissues compared to paratumor tissues	*MYH9*	Suppresses cell proliferation, migration, invasion, and EMT in vitro, and tumor growth and metastasis in vivo	[233]
miR-6126	Downregulated in EOC tissues compared to normal ovarian tissues; inversely correlated with tumor progression and positively associated with survival	*ITGB1*	Inhibits cell invasion, migration, and tube formation in vitro and angiogenesis and tumor growth in the orthotopic xenograft mouse model	[234]
miRNAs down-regulated in secreted exosomes and circulating body fluids				
miR-125a	Downregulated in serum of EOC patients compared to healthy controls	*GALNT14* *ARID3B*	Inhibits cell proliferation, invasion, and EMT in vitro	[235,236]
miR-125b	Downregulated in tissues and serum of EOC patients compared to adjacent normal ovarian tissues and serum of healthy control respectively; negatively associated with stage and lymph node and distant metastasis	*SET* *GAB2*	Inhibits cell invasion, migration, and EMT in vitro and metastasis in vivo	[113,237,238]
miR-212	Downregulated in EOC tissues and serum compared to paired adjacent normal ovarian tissues and healthy controls respectively; negatively correlated with tumor stage and metastasis	*HBEGF*	Inhibits cell proliferation, invasion, and migration in vitro	[239]
miR-122	Downregulated in serum of serous EOC patients compared to benign controls	*P4HA1*	Inhibits cell invasion, migration, and EMT in vitro, and intraperitoneal metastasis in vivo	[127,240]
miR-148a	Downregulated in plasma of EOC patients compared to healthy controls; negatively associated with tumor grade, stage, lymph node metastasis, and poor survival	ND	Inhibits cell proliferation, invasion, and migration in vitro	[241]
miR-199a	Downregulated in serum of EOC patients compared to normal controls; negatively associated with tumor grade, lymph node and distant metastasis, and poor survival	ND	ND	[128]
**miRNAs with both pro- and anti-metastatic effects**				
miR-9	Upregulated in metastatic EOC tissues compared to paired primary EOC tissues	*CDH1*	Promotes cell migration, invasion, and EMT in vitro	[242]
	Downregulated in recurrent serous EOC tissues compared to primary EOC tissues	*TLN1*	Inhibits cell proliferation, migration, and invasion in vitro	[243,244]
miR-141	Upregulated in EOC tissues compared to normal ovarian tissues, and serum of EOC patients compared to healthy controls; positively associated with tumor stage, lymph node metastasis, and poor survival	*KLF12* *SIK1* *MAPK14*	Promotes proliferation, anoikis resistance, and survival in vitro, and tumor growth and metastasis in vivo	[126,245,246]
	Downregulated in integrated mesenchymal subtype of EOC compared to normal ovarian epithelial tissues	ND	Inhibits cell migration, invasion, and EMT in vitro	[222,247]
miR-200a	Upregulated in EOC tumors compared to normal ovarian tissues and in serum of EOC patients compared to healthy controls; positively associated with aggressiveness, late stage, and lymph node metastasis	*PTEN* *MAPK14*	Promotes cell migration and invasion in vitro	[126,246,248]
	Downregulated in vasculogenic mimicry positive EOC tissues compared to vascular mimicry negative ovarian tissues and in integrated mesenchymal subtype compared to ovarian normal epithelial tissues; negatively associated with stage, ascites, and metastasis and positively correlated with patient survival	*IL8* *CXCL1* *EPHA2*	Inhibits tube formation, vasculogenic mimicry, and cell invasion in vitro, and angiogenesis and metastasis in vivo	[222,249,250]
miR-200b	Upregulated serum of EOC patients compared to healthy controls; positively associated with tumor stage, lymph node metastasis, and poor survival	ND	ND	[126]
	Downregulated in vasculogenic mimicry positive EOC tissues compared to vascular mimicry negative ovarian tissues; negatively associated with stage, ascites, and metastasis and positively correlated with patient survival	*IL8* *CXCL1*	Inhibits tube formation, vasculogenic mimicry, and cell invasion in vitro, and angiogenesis and metastasis in vivo	[249]
miR-200c	Upregulated in SOC tissues and serum of EOC patients compared to heathy controls; associated with tumor stage, lymph node metastasis, and poor survival	ND	ND	[126,251]
	Upregulated in EOC tumors compared to normal ovarian tissues; inversely associated with tumor stage and lymph node metastasis	*ZEB2* *TUBB3*	Inhibits cell migration and invasion in vitro	[252,253]
miR-203	Upregulated in EOC tissues compared with adjacent normal tissues	*PDHB*	Promotes cell proliferation, migration, and glycolysis in vitro, and tumor metastasis in vivo	[118]
	Downregulated in SOC tissues compared to adjacent normal ovarian tissue; positively associated with patient survival	*SNAI2* *BIRC5*	Inhibits cell proliferation, invasion, migration, and EMT in vitro, and tumor growth in vivo	[119,254]

ND: not determined.
